# Different fixation pattern for thoracolumbar fracture of ankylosing spondylitis: A finite element analysis

**DOI:** 10.1371/journal.pone.0250009

**Published:** 2021-04-09

**Authors:** Tianyu Zhang, Yanhua Wang, Peixun Zhang, Feng Xue, Dianying Zhang, Baoguo Jiang

**Affiliations:** 1 Department of Traumatic Orthopaedics, Peking University People’s Hospital, Beijing, China; 2 Institute of Trauma and Nerve Regeneration, Peking University People’s Hospital, Beijing, China; 3 Department of Orthopaedics, Peking University Binhai Hospital, Tianjin, China; University of Vigo, SPAIN

## Abstract

The objective of this study is to establish an ankylosing spondylitis (AS) thoracolumbar fracture finite element (FE) model and provide a proper posterior fixation choice from the biomechanical perspective. The ankylosing spondylitis T9-L5 FE model was built and the range of motion (ROM) was compared to previous studies. The L1 transverse fracture was simulated and was separately fixed by five different patterns. The pull force and yielding force of the screws, the von Mises stress of the internal fixation, and the displacement of fracture site were analyzed to evaluate the proper fixation pattern for thoracolumbar fracture of AS. ROM of AS model was obviously restricted comparing to the normal vertebral experimental data. All the fixation patterns can stabilize the fracture. At least four levels of fixation can reduce the von Mises stress of the internal fixation. Four levels fixation has a higher pull force than the six levels fixation. Skipped level fixation did not reduce the stress, pull force and yielding force. The kyphosis correction did not change the biomechanical load. At least 4 levels fixation was needed for AS thoracolumbar fracture. The cemented screws should be chosen in 4 levels fixation to increase the holding of the screws. The skipped fixation has no advantage. The kyphosis correction can be chosen after weighing the pros and cons.

## Introduction

Ankylosing spondylitis (AS) is a progressive inflammatory disorder that involves mainly the axial skeleton [1]. The inflammation makes the ligamentous ossification and zygapophyseal joint fusion [2], which leads to a rigid spine. Meanwhile, the inflammation causes osteopenia and osteoporosis of the vertebrae [3]. Both factors increase the risk of vertebral fracture [4]. The fracture of AS always affects all the three columns of the spine [5] and can happen under low energy damage [6]. Though the cervical fracture is the most common site in AS fracture, the thoracolumbar fracture is on its heel and occupies 20%-40% of AS fracture [7].

Surgical treatment is the first choice for AS fracture patients unless the patients cannot tolerate the general anesthesia or have severe comorbidities [8]. The posterior pedicle fixation is recommended for the thoracolumbar fractures of AS [5, 9, 10]. However, the screw loosening rate is up to 15% [1, 11] due to the rigid spine and osteoporosis.

Due to the lack of cohort or case-control study, the fixation pattern including the number of fixation levels, skipping level fixation [10], and kyphotic deformity correction [7] for the AS thoracolumbar fractures were still in controversial [12]. Finite element (FE) analysis can help to deal with this problem by providing the biomechanical evidence under multidirectional loading conditions.

This study was to provide biomechanical evidence for a proper posterior implant choice of the AS thoracolumbar fractures by the FE analysis. We are supposed to answer the questions as follows: (a) how many levels were enough for AS thoracolumbar fracture fixation; (b) whether skipping the level fixation is supported; and (c) should the kyphosis be corrected in the fracture treatment.

## Material and methods

### Intact model

**Fig 1:**
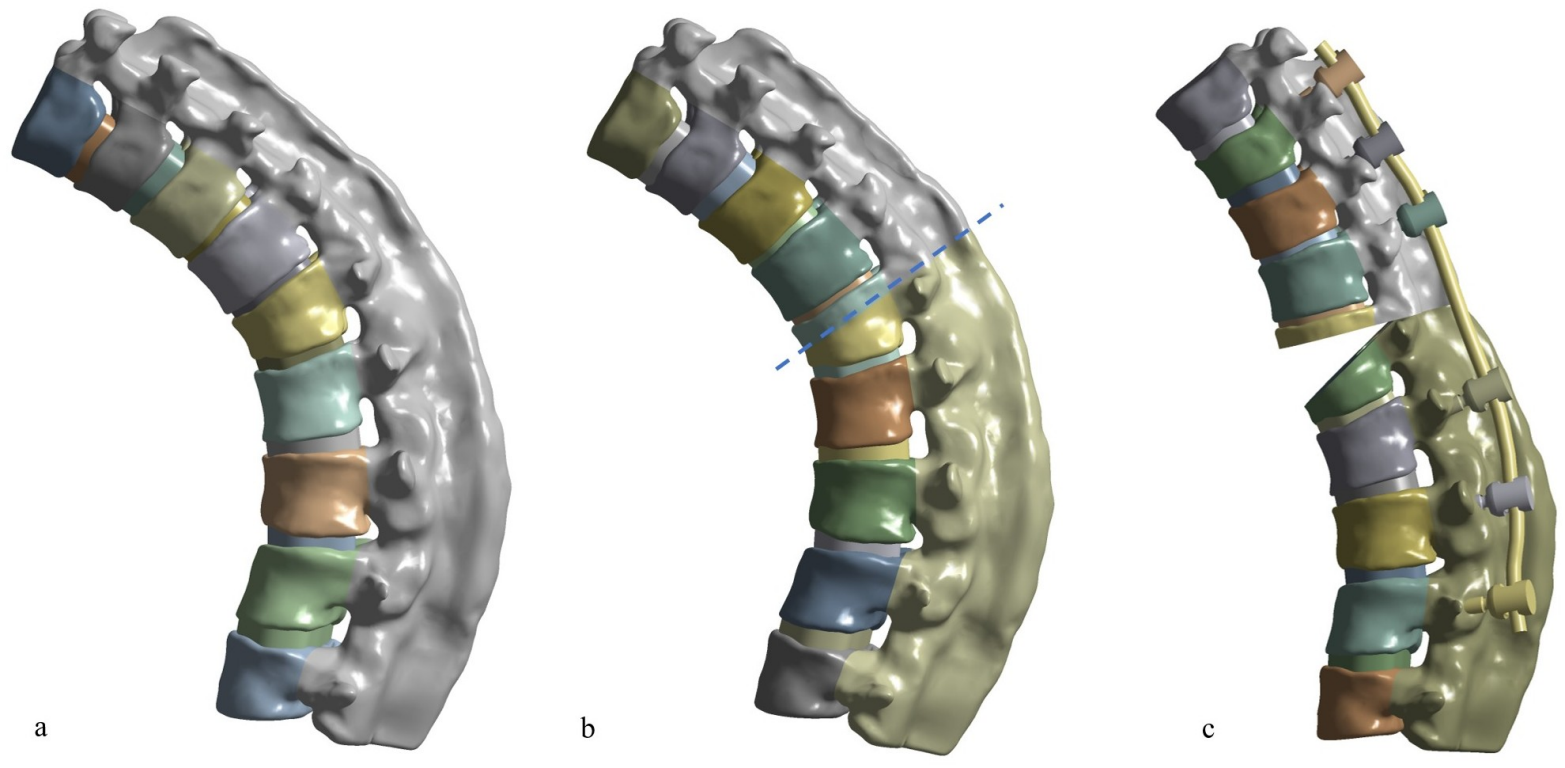
The intact AS model (a), the L1 fracture simulation (b), the 30 degrees kyphosis correction (c).

The Ethics Review Committee of Peking University People’s Hospital approved this study with the number of 2020PHB072. The patient was consented and the written consent was obtained. The data were analyzed anonymously. A T9-L5 segments FE model was developed from 6 mm-thick computed tomography (CT) of an ankylosing spondylitis 46-year-old male (height: 160cm, weight: 65kg) by software Mimics 21.0 (Materialise, Leuven, Belgium). The T9-L5 vertebrae were constructed as a whole. The cobb angle of T9-L5 was 60.9°. The vertebrae were composed of 4 mm thickness cortical outer layer [13] and inner cancellous bone [3]. The zygapophyseal joints were fused [2]. The interspinous ligaments, the supraspinal ligament, and the ligamentum flavum were defined as ossification [14]. Studio Geomagic 2017 (3D Systems, Rock Hill, SC, USA) was used to produce a more elaborate 3D model. The intervertebral discs were separately built by SolidWorks 2017 (Simulia, USA) because their contours cannot be acquired by the CT scan. The volume ratio of the nucleus pulposus to the annulus fibrosus was set to 4:6 [15]. A cortical ring was added to the outer of the intervertebral disc with a thickness of 4 mm to simulate the ossification [16]. The model comprised 9 vertebrae and 8 intervertebral discs (Fig 1a).

### Fracture model

The vertebra was cut off to simulate the vertebral traverse fracture. The fracture line came across the three columns of L1 vertebra [7, 12] (Fig 1b). Surface to surface contact friction coefficient was defined as 0.46 between the two resected extremities [17, 18].

### Fixation model

The diameter of the screw was 6.5 mm and the length of the screw were 45 mm. The diameter of the connecting rod was 6 mm. All the screws were properly placed into the pedicles using the herringbone crest vertex technique.

**Fig 2:**
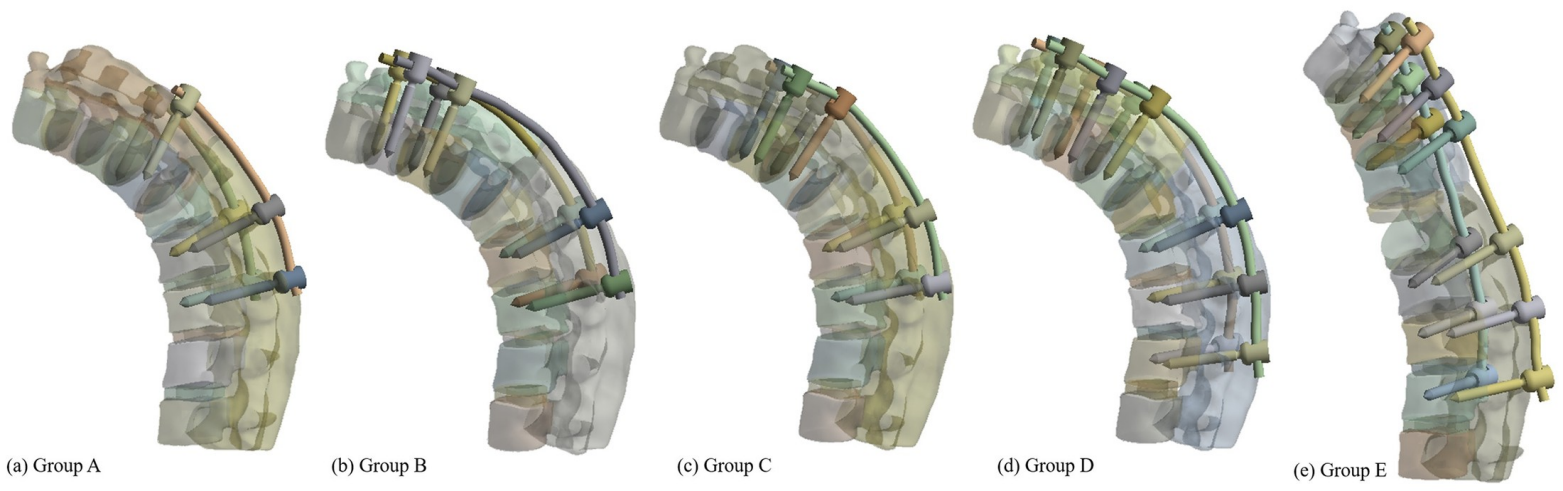
The fixation patterns and angle correction model. (a) Group A bilateral fixing T12, L2, L3. (b) Group B bilateral fixing T10, T11, L2, L3. (c) Group C bilateral fixing T11, T12, L2, L3. (d) Group D bilateral fixing T10, T11, T12, L2, L3, L4. (e) Group E 30 degrees kyphosis correction and bilateral fixing T10, T11, T12, L2, L3, L4.

The lateral views of the different fixation patterns were shown in Fig 2. All the fixation methods were bilateral fixation and the fractured vertebrae were free from screw fixation. Model A: one level upper and two levels below the fractured vertebrae were fixed. Model B: the upper one vertebra of the fractured vertebra was skipped and T10, T11, L2, L3 vertebrae were fixed. Model C: the two levels upper and two levels below fractured vertebra were fixed. Model D: the three levels upper and three levels below fractured vertebra were fixed. The Model E: a posterior osteotomy was performed and 30 degrees of kyphosis angle was corrected. Then, the three levels upper and three levels below fractured vertebra were fixed (Fig 1c).

The contacts of screws and bones, screws and rods were set as bonded. There were 542,436 nodes and 270,856 tetrahedron elements contained in intact AS FE model. The material properties are shown in Table 1.

**Table 1:** Material properties used in the finite elements models.

	Young’s Modulus (MPa)	Poisson’s ratio	References
Cortical bone	12000	0.3	[19–21]
Cancellous bone (Osteoporosis)	100	0.2	[19–21]
Posterior bone	3500	0.25	[20]
Annulus ground	4.2	0.45	[21]
Nucleus pulposus	0.2	0.49	[21]
Calcification of the annulus fibrosis	12000	0.3	[16]
Implant Properties	110000	0.3	[16, 22]

### Loading conditions

In order to compare the range of motion (ROM) of intact AS model with the ROM of normal vertebra in biomechanical experiment, a 7.5 Nm moment and a compression 150N force [22, 23] were applied at the center of the superior surface of T9. The inferior surface of L5 was immobilized. The ROM of T9-L5 was calculated and the ROM of the corresponding segment was compared with the previous experiment studies (Table 2). For testing the fixation capacity, a compressive load of 400 N force and a 10 Nm moment were applied in all fixation patterns [23–25]. Six loading conditions including flexion, extension, left bending, right bending, left axial rotation, and right rotation were simulated. Ansys workbench 17 (ANSYS Inc., PA, USA) was used to set the material properties and simulate the loading conditions.

**Table 2:** Comparison of the range of motion between the intact ankylosing spondylitis model and models from previous studies.

ROM	ROM of T9/L5(°)	ROM of L1/2 (°)		ROM of L2/3 (°)		ROM of T12/L2 (°)	
	Present study	Present study	Yamamoto [27], (mean±SD)	Present study	Yamamoto [27], (mean±SD)	Present study	Pflugmacher [28], (mean±SD)
Flexion	2.28	0.92	5.8±0.6	1.58	6.5±0.3	1.78	5.3±1.0
Extension	2.26	0.72	4.3±0.5	1.28	4.3±0.3	1.88	5.7±1.0
Left lateral bending	0.62	0.05	5.2±0.4	0.07	7.0±0.6	0.1	4.3±0.6
Right lateral bending	0.5	0.08	4.7±0.4	0.9	7.0±0.6	0.16	4.3±0.6
Left axial rotation	1.29	0.64	2.6±0.5	0.44	2.2±0.4	0.82	2.1±0.5
Right axial rotation	0.95	0.51	2.0±0.6	0.23	3.0±0.4	0.71	2.1±0.5

### Assessment indexes

The pull force, yielding force, von Mises stress of the internal fixation, and displacement in the fracture site were calculated under the different loading conditions. Pull force and yielding force were used to judge the possibility of screw loosening [26]. The pull force was measured by the force along the direction of the screw. The force resulting in the screw pull-out trend was recorded in comparing the maximum pull force. The yielding force was measured by the force along the direction of rods and the direction of force (positive and negative) was both recorded. The maximum pull and yielding forces among the screws in different loading conditions were compared. The von Mises stress distribution of the screws and rods was obtained to judge the risk of failure of the internal fixation. Displacement of the fracture site was used to estimate the fixation stability. The relative displacement of the fractured vertebral body posterior margin was recorded. The displacement distance was divided into vertical displacement and horizontal displacement [16]. The vertical displacement was used to judge the fixation stability. The horizontal displacement was considered as the risk of spinal cord injury. The proper fixation pattern needs to fulfill low pull force, low yielding force, low von Mises stress of internal fixation, and high fixation stability. There was no statistical analysis for only one subject.

## Results

### Comparison of the ROM with the previous studies

The ROM of T9-L5 in the AS model was 2.28° in flexion, 2.26° in extension, 0.62° in left bending, 0.5° in right bending, 1.29° in left rotation, and 0.95° in rotation under a 7.5 Nm moment and a 150N compression force. The ROM of AS model was decreased comparing to the ROM of the normal spine biomechanical experiments in the corresponding segment (Table 2) [27, 28].

### Von Mises stress distribution of the internal fixations

The maximum von Mises stresses were 653.36 MPa in group A, 282.18 MPa in group B, 239.02 MPa in group C, 221.21 MPa in group D, and 267.79 MPa in group E (Table 3). The internal fixation of AS fracture suffered from higher von Mises stress than the internal fixation of normal vertebra fracture under the same fixed levels [22, 23].

**Table 3:** The maximum pullout force, maximum yielding force, maximum von Mises stress of the internal fixation, and maximum displacement in the fracture site from all loading conditions were presented.

Group	Maximum pull force (N)	Maximum yielding force (N)	Maximum von Mises stress of internal fixation (MPa)	Maximum Horizontal displacement (mm)	Maximum Vertical displacement (mm)
A	154.86	404.93	653.36	0.367	0.210
B	208.97	347.79	282.18	0.316	0.170
C	224.58	325.94	239.02	0.304	0.167
D	138.89	260.38	221.21	0.246	0.175
E	149.32	318.00	267.79	0.254	0.343

**Fig 3:**
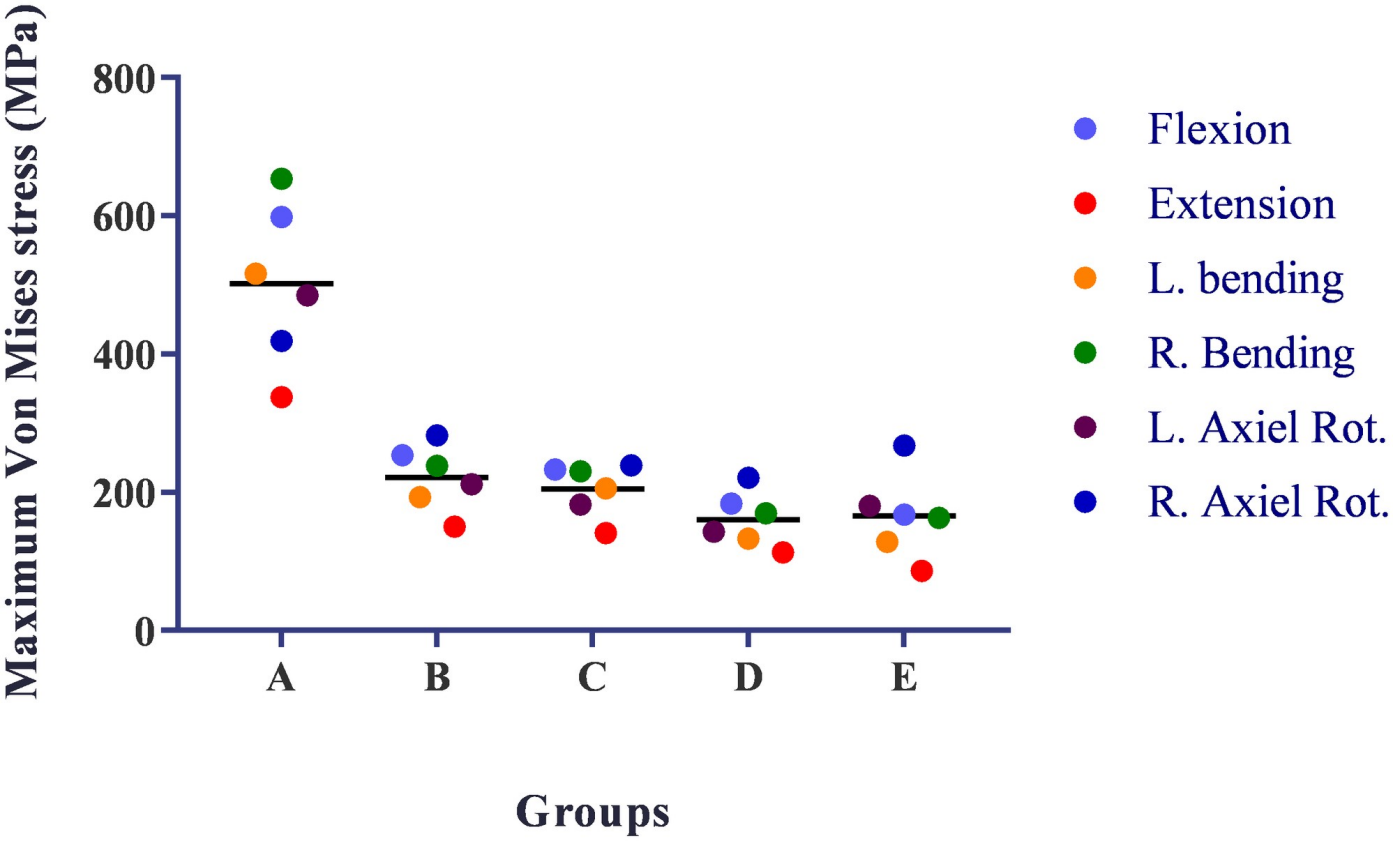
The maximum von Mises stress (MPa) on the screws and rod in different fixation models under different loading conditions. L. = Left, R. = Right, Rot. = Rotation.

The maximum stress on internal fixation was more likely to happen in the flexion and bending conditions. The maximum von Mises stress was approximately 2.5 times more in group A than other groups. There was no obvious difference between group B, C, D, and E in maximum stress (Fig 3). According to these results, the fixation pattern of group A had the risk of internal fixation failure.

### Pull force and yielding force

**Fig 4:**
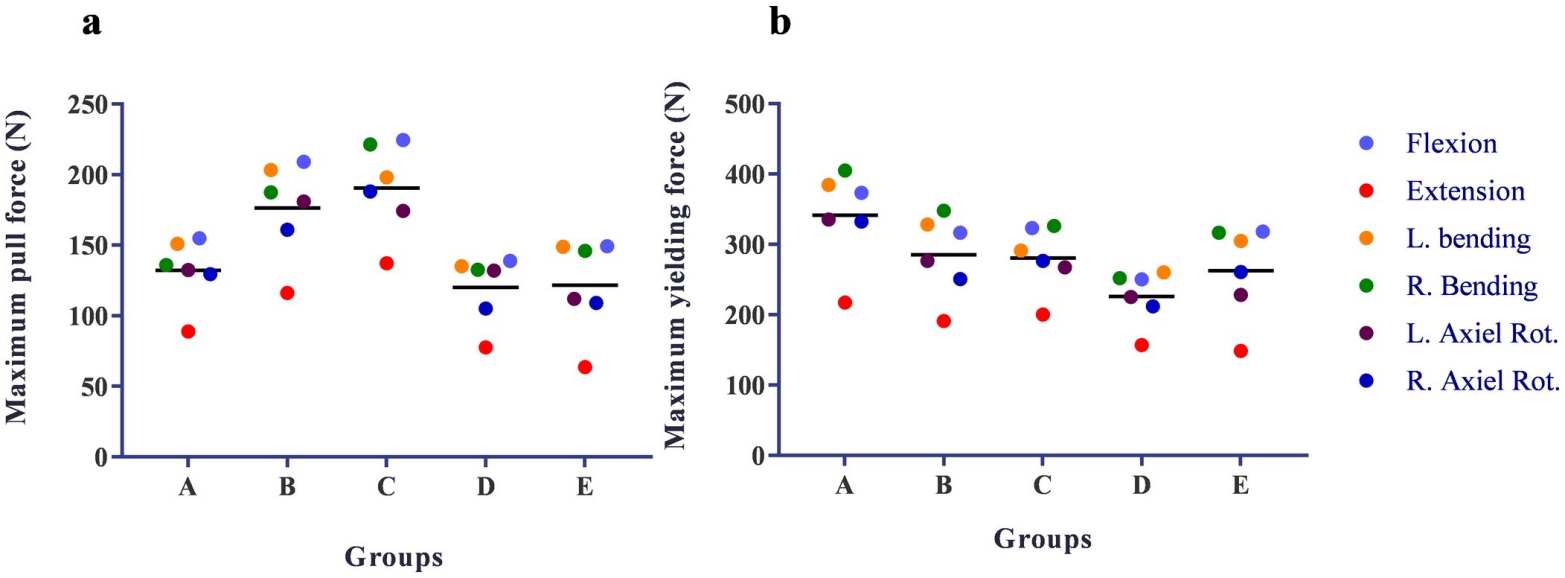
The maximum pull force (N) (a) and yielding force (N) (b) in different fixation models under different loading conditions. L. = Left, R. = Right, Rot. = Rotation.

The maximum pull forces and maximum yielding forces of all groups were listed in the Table 3. The screws suffered the max pull and yielding force under the bending and flexion conditions. Group C had the highest pull force though the fixation levels were more than group A. Group D had the minimum pull force and yielding force. The skipped levels fixation of group B did not obviously reduce the pull force and yielding force comparing to the same fixation levels of group C. Kyphosis correction in group E did not obviously change the pull force and yielding force comparing to the group D (Fig 4).

#### Displacement of the fracture site

**Fig 5:**
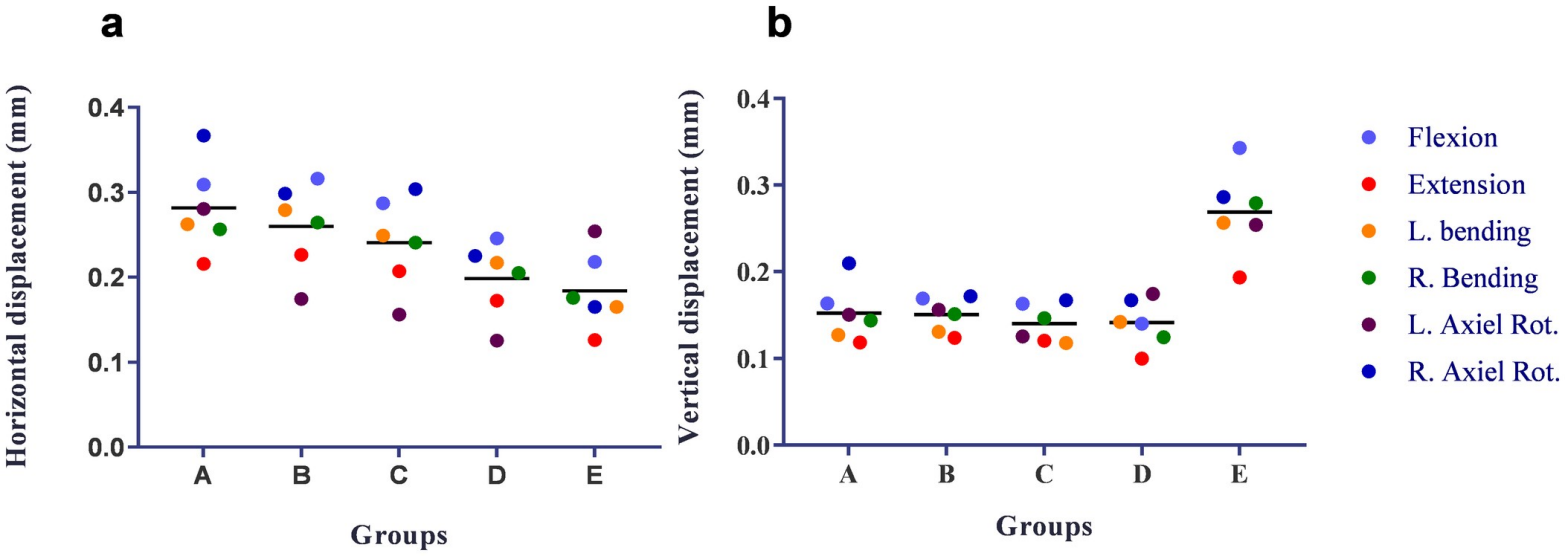
The displacement of the two ends of the fractured vertebra in different fixation models under different loading conditions. Horizontal displacement (mm) (a), Vertical displacement (mm) (b). L. = Left, R. = Right, Rot. = Rotation.

The maximum horizontal displacement and maximum vertical displacement were listed in Table 3. The fracture site horizontal displacement decreased with the increasing of the fixation levels. The group E had the highest vertical displacement. However, all the displacements were less than 0.5 mm, which could be regarded as a strong fixation (Fig 5).

## Discussion

Inflammation and new bone formation were the main characteristics of the AS, which increase the risk of fracture [1]. To our knowledge, there is a lack of biomechanical study about the AS thoracolumbar fracture. This article presented a finite element AS thoracolumbar fracture model to guide the posterior pedicle fixation. The main outcomes were: (a) In thoracolumbar fracture of AS, the internal fixation suffered more von Mises stress than normal spine thoracolumbar fracture [23]. (b) In thoracolumbar fracture of AS, at least 4 levels fixation were recommended. (c) There is no advantage of skipped level fixation on biomechanics. (d) The kyphosis correction did not change the biomechanical load.

Due to the ossification of the ligaments and zygapophyseal joints, the ROM of the AS spine was restricted comparing to the normal spine. This character was well simulated by our AS model.

### Characteristics of the AS thoracolumbar fracture fixation

The rigid spine and transverse three-column fracture produced a higher von Mises stress on the internal fixation compare to the normal vertebra thoracolumbar fracture [22, 23]. More fixed levels were needed in AS thoracolumbar fracture. Stress concentrating on the internal fixation can obviously be reduced when the fixed levels were no less than four in our model. Therefore, at least four levels of fixation were recommended, which was consistent with the practical experience [1, 8].

### Pull force and yielding force

The pull force and yielding force were calculated to evaluate the possibility of fixation loosening, which happened in 15% of patients after posterior fixation [29]. The vertebral osteoporosis extent, screw length, screw diameter, screw core diameter, and bonding pattern of the vertebra and screw can all influence the pull force that screw can tolerate [20, 30]. In actual clinical settings, the fatigue could also lead to the loosening of the screws under the multiple loading. Based on the above reasons, direct comparison of the FE model result with the previous biomechanical experience research was inappropriate. Therefore, our study compared the relative force among fixation patterns to find a balance between the risk of screws loosening and surgical trauma. Due to the higher pull force in 4 levels fixation comparing to the 6 levels fixation, the cement augmented screws are recommended in 4 levels fixation.

### Levels fixation

Excessive screws may increase the unnecessary screws cost and the risk of misplacement [31]. Moreover, the placement of screws is difficult for AS patients because of soft tissue calcification and vertebral bone hyperplasia [5]. Therefore, reducing the fixation segments are needed under firm fixation.

Von Mises stress of 4 levels fixation was at least twice less than 3 levels fixation. However, the 4 levels fixation had a higher pull force comparing to the 6 levels fixation. As a result, 4 levels with cemented screws [10] or 6 levels with ordinary pedicle screws were recommended in AS thoracolumbar fracture. Other strategies can be chosen to reduce the strain without increasing the fixation segment, such as reducing the stiffness of the rods [10].

### Skipped level fixation

The skipped fixation has been extensive applied in long bone fracture, which increases the working distance of the internal fixation [32]. The same principle was advised to apply to the spine fracture of AS by leaving at least one vertebral body adjacent to the fracture vertebra [10, 16]. We simulated this fixation pattern by FE analysis. Under the same fixed levels, the skipped level fixation did not present superiority in internal fixation stress, pull force, and yielding force. At the same time, the skipped level fixation needed incision extended. Consequently, the skipped level fixation was not advised to be performed in thoracolumbar fracture of AS.

### Kyphosis correction

The study in kyphosis correction of thoracolumbar AS fracture was limited. Werner summarized the case series studies and found that the deformity correction could increase the complications [33]. However, the kyphosis correction has not been studied in the aspect of biomechanics. Thirty degrees kyphosis correction was performed in our model and it did not obviously change the stress of internal fixation and the risk of screws loosening. On the one hand, the reduction of the cobb angle changed the stress conduction and decreased the stress distribution on the internal fixation. On the other hand, the kyphosis correction caused the absence of anterior column supporting and leaded to more stress. These two effects counterbalanced each other. Hence, 30 degrees of kyphosis correction had no obvious influence in the aspect of biomechanics. Surgeons are supposed to balance the advantage of restoring sagittal balance and the risk of complications when they make decisions.

### Limitation of this study

Due to no biomechanical research on the cadaveric model of AS spine [16], direct validation was impossible. We compared the ROM of normal spine with our FE model and the model was based on logical assumptions to simulate reality as close as possible. Second, this model did not simulate the muscles of the spine, which may influence the spinal stability. Nevertheless, the FE model still can reflect the biomechanical behavior of the AS thoracolumbar fracture fixation.

## Conclusions

The AS thoracolumbar fracture fixation suffered higher stress than normal spine thoracolumbar fracture. At least 4 levels fixation was needed for AS thoracolumbar fracture. The cemented screws should be chosen in 4 levels fixation to increase the holding of the screw. The skipped level fixation had no advantage. The kyphosis correction could be chosen after weighing the pros and cons.

## Abbreviations

AS: Ankylosing spondylitis
FE: Finite element
L.: Left
R.: Right
ROM: range of motion
Rot.: Rotation
